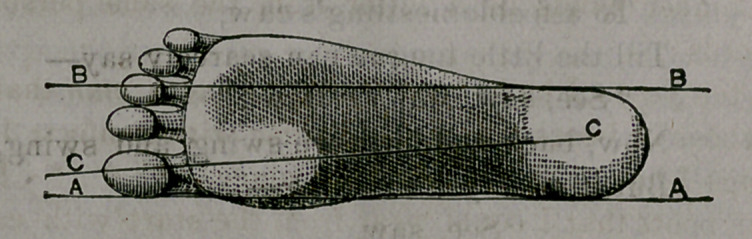# An Article by Dr. Hall

**Published:** 1876-07

**Authors:** 


### AN ARTICLE BY DR. HALL.

(We find the following article among the manuscripts prepared for the pages of the JOURNAL by our late lamented associate, Dr. W. W. Hall, and accordingly give it a place.)

### A DOUBLE CRITICISM.

☞ One of our readers criticises the editor of the JOURNAL and Mr. Joel McComber in the same breath. Some time ago we received a letter from a gentleman in Ohio, complaining of us for our favorable mention of Mr. McComber's excellent and improved boots, shoes, and lasts, and commenting very unfavorably on the results of his own attempts to secure a comfortable fit by the McComber method. The letter said that if what we had published was merely advertising matter furnished by Mr. McComber, the writer would not complain ; but if the expressions were really our own opinions, he must take exceptions to them. The writer then details his efforts to secure a pair of the McComber boots. He says that the first two pairs were too large, and the next pair was too small, and that he was dissatisfied with them. We saw Mr. McComber soon afterward and asked him about the case. He stated the facts about as our correspondent had stated them, and then looked up the diagram of our correspondent's foot. It was plain to see that it was not a natural-shaped foot. We made a tracing of it with the result shown below.

The outline exactly represents our correspondent's foot ; the other cut represents a natural and healthy one. One shows a foot badly broken and distorted, as will be seen. It ought not to be doubted, that this person has suffered long, from badly-formed shoes, in order to account for the malformation so apparent here. But he makes no such admission to us. From all that we can gather by his remarks, his feet are as perfect as anybody's. His letter is made up mainly of complaints of the McComber system, and of the JOURNAL for recommending it. Still, he is willing to forgive us if we will deny saying the favorable words which have appeared in these pages, except as paid puffs, and not out of the fullness of our own heart.

We are sorry we must seem disobliging, but the truth must be told in justice to Mr. McComber. That gentleman has never paid us one sixpence for our editorial comments on his system. He has occasionally made his business known in our advertising pages, and for that service he has paid our publishers, we have no doubt. But our editorial comments upon his method and his goods are our own views, uttered in type precisely as we are in the practice of uttering them by word of mouth to our patients and friends. When we say that McComber's system is the best we ever saw, we speak what we know to be the truth; and when we say we think it is the best in the world, we state what we believe. The fact that our Ohio friend found his first two pairs of McComber boots too large, and the third pair too small, does not prove us in error by any means. We suppose such an accident may happen under any system. The size is not the point; it is the *principle involved* that we have heretofore discussed. We say the principle is right, that it conforms to the anatomy of the foot, and that no other system does. One pair, or one thousand pairs of boots may be made too large, or too small, so that they are too loose for elegance, or too tight for comfort, yet the system remains unsurpassed. We have seen hundreds of pairs of McComber's boots and shoes on the feet of friends and acquaintances of ours, and have yet to see the first misfit or hear the first complaint other than this, except in two instances—one where a lady frankly admitted that she preferred pinched toes and corns to a proper-sized shoe, and the other being the case of a party to whom we will allude presently.

There are some points about the case of our Ohio friend which deserve especial attention. He would have us infer that his feet are perfect. In fact, he says that another shoemaker has fitted him satisfactorily. But Mr. McComber hands us a letter from the same person in which we find these words :

"For ten years I have been trying to find a man that could make me a pair of shoes or boots that I could wear with comfort; but alas! in all my searchings I have not yet found the place. * * * If I could ever get a pair of boots that I could wear from the start with ease and comfort, I would think I had almost found a heaven on earth."

So, then, the diagram which says he has bad feet, distorted feet, feet which have been tortured out of all shape and comfort, tells the trnth. That he never succeeds in finding new boots which are easy, is abundantly testified to. Yet he conceals these facts from us, and would have us denounce that great boon to suffering humanity—the McComber patent last—because, by some accident, or for some unaccountable reason, he receives two pairs of boots which are, in his opinion, too large, and another too small. Perhaps if the larger pair had been worn for a few days they would have been found all right.

Another curious fact is this. By the printed letter-head used by our correspondent we find that he is interested in the shoe business. We will hazard a guess that he does not use the McComber last and that he wishes he possessed the right so to do !

The other complainer, of whom we spoke a few lines back, was also a shoemaker. He had endeavored to buy the privilege of using McComber's lasts jointly with the old-fashioned strips of wood called lasts on which boots and shoes are usually made. Mr. McComber refused to license him unless he would abandon the old lasts. As he was an old carpenter in leather, and had thousands of pairs of venerable, patched-up blocks for making cripples with, he declined to make fire-wood of them, and so he was not licensed. He complained bitterly. He told us that McComber's boots fitted nicely, and were very comfortable at first, and then got uncomfortable. We told him that the comparison "easy as an old shoe" was a proverb, and we were willing to take our chances with shoes which were easy at first. Besides, we had worn Mr. McComber's boots when old as well as when new, and they had not only proved delightfully easy in the beginning, but had so continued to the end ; never sprawling out or spreading, the upper beyond the sole. In fact, we have on, at this moment, a pair of Congress gaiters made by Mr. McComber, and worn by us for more than a year, which are now as perfect as ever, though they have been tapped once or twice. They have not only kept in shape and given us perfect comfort, but our feet, before badly distorted, have improved in shape through their influence. We paid Mr. McComber $10 cash for them, and we don't know that we ever spent money which has done us more good.

Is it not curious that while we have heard thousands of encomiums upon McComber's system by physicians and others, and while hundreds of letters have reached us commending the improved goods, and thanking us for recommending them, the only complainers have been two shoemakers and a silly girl who had hardly breath with which to complain, so tightly was she laced? We have recommended Mr. McComber's excellent system, and have probably been the means of increasing his business. If so, we are glad. We have spoken well of the Health Food Company, and the very valuable work which they have done and are doing for the human race. They say we have helped them. We trust so. Good things do not thrive without much labor, and great expenditure of time and money. We are happy when we can show the world how to improve its condition, and we shall continue to encourage worthy objects so long as our life is spared.

## Figures and Tables

**Figure f1:**
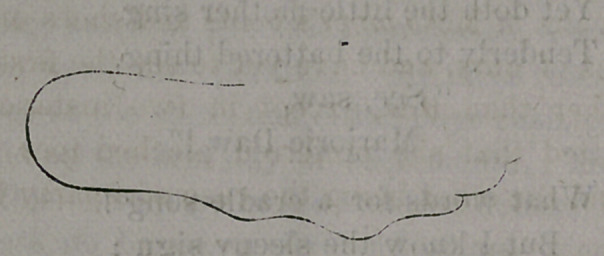


**Figure f2:**